# Knowledge Production and Learning for Sustainable Landscapes: Seven Steps Using Social–Ecological Systems as Laboratories

**DOI:** 10.1007/s13280-012-0367-1

**Published:** 2013-03-10

**Authors:** Per Angelstam, Marine Elbakidze, Robert Axelsson, Malcolm Dixelius, Johan Törnblom

**Affiliations:** 1 Faculty of Forest Sciences, School for Forest Management, Swedish University of Agricultural Sciences, PO Box 43, 730 91 Skinnskatteberg, Sweden; 2 Faculty of Forest Sciences, School for Forest Management, Swedish University of Agricultural Sciences, PO Box 43, 739 21 Skinnskatteberg, Sweden; 3 DiXit International, PO Box 24, 139 03 Stavsnäs, Sweden

**Keywords:** Ecosystem services, Sustainability science, Social learning, Governance, Case study, Transdisciplinary research

## Abstract

There are multiple challenges regarding use and governance of landscapes’ goods, functions and intangible values for ecosystem health and human well-being. One group of challenges is to measure and assess principal sustainability dimensions through performance targets, so stakeholders have transparent information about states and trends. Another group is to develop adaptive governance at multiple levels, and management of larger geographical areas across scales. Addressing these challenges, we present a framework for transdisciplinary research using multiple landscapes as place-based case studies that integrates multiple research disciplines and non-academic actors: (1) identify a suite of landscapes, and for each (2) review landscape history, (3) map stakeholders, use and non-use values, products and land use, (4) analyze institutions, policies and the system of governance, (5) measure ecological, economic, social and cultural sustainability, (6) assess sustainability dimensions and governance, and finally (7) make comparisons and synthesize. Collaboration, communication and dissemination are additional core features. We discuss barriers bridges and bridges for applying this approach.

**Electronic supplementary material:**

The online version of this article (doi:10.1007/s13280-012-0367-1) contains supplementary material, which is available to authorized users.

## Introduction

The sustainable development (SD) discourse began in the 1980s (see Baker 2006; Dresner 2008). Since then a range of international and national policies have been formulated related to ecologically, economically, and socially sustainable use of natural resources, as well as adaptive management and governance of them (UNECE 1998; Council of Europe 2000; FAO 2003; European Commission 2004; Forest Europe 2011). Additionally, cultural sustainability is emerging as a fourth pillar (Chan et al. 2012; Daniel et al. 2012; Axelsson et al. 2013a). These policies apply to the delivery of natural resources in terms of food, wood, fibers, and energy, and also for benefits such as human well-being, regulation of ecological processes, protection of habitat for species, and maintenance of cultural values (Merlo and Croitoru 2005; Kumar 2010). The vision is thus based on sustainable landscapes, including natural systems and space as well as human systems and place (Haines-Young 2000; Antrop 2006). The ecosystem approach (see Österblom et al. 2010) and ecosystem service concept capture this by linking ecosystems to societal benefits (MEA 2005; Kumar 2010; Norgaard 2010).

Nevertheless, use and management of landscapes are often unsustainable (e.g., Butchart et al. 2010), and stakeholders act independently of each other (Young 2013). Simultaneously, multiple sectors with management responsibility at different societal levels of governance are challenged with sharing power and improving collaboration among stakeholders in social–ecological systems (e.g., Adger and Jordan 2009; Axelsson et al. 2013b). Additionally, there is a need to consider risks and uncertainties related to continually evolving expectations from society (Innes and Hoen 2005; Zaremba 2012), variable market demands and economic crises (Barnes 2006), and to climate change (Johnston and Williamson 2007). Dealing with all of this complexity is the paramount management and governance challenge for civil, private, and public sectors (Gunderson et al. 1995; Franklin and Blyton 2011; Komiyama et al. 2011).

There are several gaps between policies for natural resource governance and management and what is practiced on the ground. These gaps can be divided into two groups (Lee 1993). The first is related to the key challenge of incorporating multifaceted values into management and governance (Kareiva et al. 2011; Axelsson et al. 2013a). For example, there are gaps between the way landscapes are described and monitored in practice (e.g., focus on material products at the stand scale) and what ought to be the case following policy (e.g., also including economic non-use values, ecological and socio-cultural dimensions at multiple scales). The second group is related to the limited understanding on how to develop locally and regionally adapted multi-level and multi-stakeholder governance systems (Adger and Jordan 2009; Sandström et al. 2011; Young 2013).

Sustainable Development (SD) is a societal process towards sustainability, and requires both a gyroscope and a compass (sensu Lee 1993). While the gyroscope is about societal steering as the totality of formal and informal types of governance at multiple levels (e.g., Baker 2006), the compass is about providing transparent knowledge about the states and trends of different sustainability criteria (Norton 2005). The insight that this requires novel approaches to knowledge production (Gibbons et al. 1994) is not new. Odum (1959) highlighted the need to understand both the extreme ability of people to control and influence their surroundings (i.e., the ecological system), and that humans develop culture in terms of the way people live in different areas, times and settings (i.e., the social system). Since then there has been a proliferation in the literature by scholars (e.g., Franklin and Blyton 2011; Komiyama et al. 2011), policies at multiple levels (e.g., Axelsson et al. 2013b) and donors’ visions for research (Regeringens Proposition 2012; Angelstam et al. 2013a) that address the issue of how to encourage SD as a social process and sustainability as consequences in social–ecological systems. Two fundamental conclusions are that the borders among academic disciplines need to become more porous, and that academic and non-academic actors need to collaborate using both quantitative and qualitative methods (Hirsch Hadorn et al. 2008; Komiyama et al. 2011). The term transdisciplinary research captures this (e.g., Leavy 2011). However, different disciplines use different frameworks, concepts, words and even languages to describe and analyze the complexity of landscapes as coupled social and ecological systems (Snow 1993). How can this gap be bridged in practice?

Several concepts that aim at integrating governance and management in social–ecological systems towards sustainability on the ground have appeared during the past two decades (Axelsson et al. 2011, 2013b). These concepts include international examples, such as UNESCO Biosphere Reserve (e.g., Elbakidze et al. 2013c), Model Forest (e.g., IMFN 2008), Agenda 21 (e.g., Smardon 2008), and EU Leader (Moseley 2003), and national ones such as the Polish Promotional Forest Complex (Blicharska et al. 2012). In addition there are business management concepts advocating special efforts towards sustainability on the ground, and cultural landscapes based on a long history of applying traditional knowledge in land use and local governance arrangements (Elbakidze and Angelstam 2007; Parrotta and Trosper 2012). Employing such concepts on the ground is consistent with the term landscape approach (see Axelsson et al. 2011, 2013b). However, while being a common attempt towards development it does not necessarily mean that an integrated approach for learning is achieved (Axelsson et al. 2013b). Experiences from and analyses of individual landscape approach initiatives in different phases of development (Axelsson et al. 2013b), and social–ecological systems without special initiatives, have so far been poorly utilized for learning and knowledge production for sustainable landscapes (Potschin and Haines-Young 2012). To create a structured approach for compilation, comparison and synthesis from studies of multiple social–ecological systems, or landscapes, there is need for standardized frameworks to organize findings (Ostrom 2009).

**Fig. 1:**
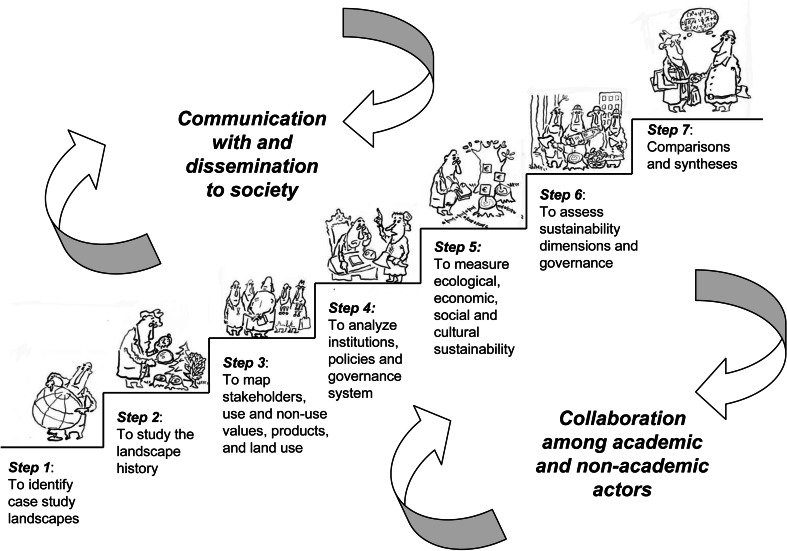
Illustration of the seven-step framework for knowledge production and learning to support the development of accounting systems for ecological, economic, and socio-cultural dimensions of sustainability, as well as adaptive management and governance (from Angelstam et al. 2007). Drawings by Leonid Kovriga

The aim of this paper is to present a new framework for integrative sustainability science in seven steps, which goes beyond interdisciplinary approaches to understand social–ecological systems in the context of ecosystem health and human well-being. Sustainability science draws upon the theories and applications of SD and landscape ecology, is use-inspired and primarily multi- and interdisciplinary (Wu 2006; Musacchio 2009; Kates 2011). By also including stakeholders of natural resource use systems as well as policy and management implementation, our focus is not only on knowledge production, but also on collaborative learning towards sustainable landscapes, i.e., transdisciplinary research. To improve the opportunity for comparative studies and meta-analyses, our approach is to collect data from multiple social–ecological systems as case studies that represent gradients in landscape history and approaches to societal steering. The selected case studies cover large areas such as entire municipalities, landowner management units or entire river catchments (see Barbour et al. 2004; Roni 2005). This approach is consistent with natural experiments sensu Diamond (1986), landscape laboratories and quasi-experiments sensu Merriam (1988), Kohler (2002), and Tyrväinen et al. (2006). First, we describe a systematic framework in seven steps (Fig. 1) with the aim to produce new knowledge for sustainable landscapes as a collaborative learning process among researchers from different disciplines, and non-academic actors. Second, we elaborate on collaboration among researchers and practitioners. Third, we stress the need for communication with, and dissemination to policy-makers and the public. Finally, based on our experiences of applying this approach in multiple European landscapes with different governance arrangements and histories, we discuss barriers and bridges to the application of problem-solving transdisciplinary research in the context of natural resource use.

## A Systematic Approach to Knowledge Production

### Seven Steps Using Multiple Frameworks and Methods

#### Step 1. Identify a Suite of Landscapes as Case Studies

Knowledge production for SD towards sustainability with multiple landscapes, i.e., social–ecological systems, as replicated case studies requires sampling in gradients that represent variation in different dimensions (Best 2009; Angelstam et al. 2011b). To cover the variation among regions in Europe’s East and West, the location of landscapes as case studies is stratified by factors that capture different aspects of landscapes as social–ecological systems (Angelstam et al. 2013c, d). The different landscape schools in geography provide important insights into the study of places and spaces (Wiens et al. 2007; Angelstam et al. 2013d). For any given ecoregion’s biophysical conditions (e.g., topography, bedrock and soils) these include (1) environmental and economic history of landscapes, and (2) system of governance, institutions, and culture (Angelstam and Törnblom 2004; see Table 1). Including countries in the entire European continent’s East and West ensures considerable variation among spaces and places (Best 2009; Angelstam et al. 2011b).

**Table 1:** Multiple landscape case studies of social–ecological systems representing particular geographical areas as spaces and their social system as places are valuable for comparative studies of how different systems of governance deliver different dimensions of sustainability (see Angelstam and Törnblom 2004; Angelstam et al. 2011b). Focusing on the European continent’s East and West, from Sweden to Russia, to apply the landscape laboratory idea, we give examples of how countries with different governance systems and political culture (columns), and with different landscape histories linked to economic development (e.g., Chirot 1989) (rows), can be used to stratify data from individual social–ecological systems, or landscapes, as case studies. The cells in the matrix contain short descriptions of the case studies, and the approximate latitude and longitude. For a list of our publications about each of them, see Electronic Supplementary Material

Landscape history	Governance system		
	“Western civilization”, west of the former Warsaw Pact	Countries in transition	“Orthodox civilization”, east of the western border of the Orthodox religion
Shorter	Ångermanälven catchment and Vilhelmina Model Forest (northwest Sweden) (64°N; 16°E)	Bialowieza forest (northeast Poland) (52°N; 24°E)	Kovdozersky Model Forest (Murmansk oblast, northwest Russia) (66°N; 32°E)
Intermediate	Bergslagen region (south-central Sweden) (60°N; 15°E)	The Carpathian Mountains in Lviv region (west Ukraine) (49°N; 23°E)	Priluzie Model Forest (Komi Republic, northwest Russia) (60°N; 49°E)
Longer	Helge å catchment and Kristianstad Vattenrike (south Sweden) (56°N; 14°E)	Roztochya Biosphere Reserve (west Ukraine) (49°N; 24°E)	Pskov Model Forest (Pskov oblast, west Russia) (57°N; 28°E)

#### Step 2. Study the Landscape History

Landscapes have been shaped by different natural and cultural disturbance regimes, with different intensities and over different time spans (Birks et al. 1988; Angelstam et al. 2013d). To understand the prerequisites for SD toward sustainability in social–ecological systems, their history needs to be analyzed (Angelstam et al. 2013b). This implies a need to consider and understand the consequences of past human use and influence in the landscape (Gunst 1989). Inspired by Worster (2005) we focus on three aspects: (1) Natural environments of the past. How did the ecosystem develop in terms of composition, structure and function? (2) Human modes of production. How did the productive technology of the social system interact with the ecosystem? (3) Perception, ideology and values. What is the role of the intangible dimensions when dealing with ecosystems?

#### Step 3. Map Stakeholders, Use and Non-use Values, Products, and Land Use

To understand the current state and trends of ecological, economic, social and cultural dimensions of SD, and governance systems, it is important to consider all stakeholders involved with the use, management and governance of natural resources in landscapes. Several sub-steps should be taken. The first is to map landscape stakeholders of different categories (Elbakidze et al. 2012; Axelsson et al. 2013b). One approach is to survey stakeholders according to: (i) the sector which they represent, that is civil, private or public; (ii) their level of activity, that is at local, regional, constitutional, and international level. A second approach is to describe landscapes’ use and non-use values, and the products derived (cf. Merlo and Croitoru 2005; Richnau et al. 2013; Elbakidze et al. 2013a). A third way is to identify and analyze the types of land cover and land use related to use and non-use values (Merlo and Croitoru 2005). This includes analyses of property and land use-rights to understand what kinds of benefits and interests are connected to each particular landscape’s different land covers (Elbakidze and Angelstam 2007; Angelstam et al. 2011b; Elbakidze et al. 2011).

#### Step 4. Analyze Institutions, Policies, and Governance System

Natural resource management and use is dependent on the societal context (Lehtinen 2006). This includes formal and informal institutions, that is rules and norms in use (Pahl-Wostl 2006), policy (Elbakidze et al. 2013c) and levels of collaboration among stakeholders at multiple levels (Elbakidze et al. 2010; Axelsson et al. 2011). There is an ongoing transition from government to governance (Rhodes 1997; Kooiman 2003), that is from government-dominated steering to shared governance incorporating stakeholders from multiple sectors. Thus, this step analyses the governance system at different levels including the investigated landscapes and its surroundings (Axelsson et al. 2013b).

A critical issue is to understand the policy visions and their corresponding ambition levels for sustainability. Such “benchmarks of sustainability” may be derived from analyses of international and national policy documents (Angelstam et al. 2011a). Regarding ecological sustainability, policy visions can be used to develop both evidence-based and negotiated performance targets for different dimensions of sustainability. Non-linear responses of species to habitat loss and certification standards exemplify this (Angelstam et al. 2013e). Biodiversity conservation ambition levels can be interpreted by comparative studies of focal species in landscapes with different histories (Roberge et al. 2008), retrospective studies (Lindborg and Eriksson 2004) and modeling (Fahrig 2002). Similarly, studies in comparative politics have found factors that affect governance and social capital (Putnam et al. 1993). However, national policy, programmatic, management or strategy documents specific to the studied landscape may be different than international ones. These may thus not be reflected or even shared at a local or regional level. Analysis at this level should match the scale of investigation, reflecting the specific challenges, values and opportunities of a particular case study.

#### Step 5. Measure Ecological, Economic, Social, and Cultural Sustainability

The aim of this step is to develop and apply methods to measure the ecological, economic, social and cultural states of the selected social–ecological system. This means to operationalize policy principle’s different criteria and indicators by identifying and using measurable verifier variables that reflect different spatial scales (Lammerts van Bueren and Blom 1997; Axelsson et al. 2013a; Elbakidze et al. 2013b). The biophysical, anthropogenic, and intangible landscape concepts can be used as a tool to include and bridge theories from different disciplines, and to identify verifier variables for different aspects of sustainability (Angelstam et al. 2013e; Axelsson et al. 2013a). Subsequently, states and development trends of ecological, economic, social, and cultural dimensions can be compared. Here both qualitative and quantitative methods are needed (Axelsson et al. 2013a; Richnau et al. 2013). It is, however, crucial to critically analyze whether the indicators proposed in policy processes really form state indicators, and not only response or pressure indicators (sensu Butchart et al. 2010).

#### Step 6. Assess Sustainability Dimensions and Governance

The term “policy cycle” captures the dynamic interactions among policy, governance, management and assessment in a particular field (Howlett and Ramesh 1995; Bridgman 2003; Mayers and Bass 2004). Assessment is a crucial part of the policy cycle (Weaver and Rotmans 2006; Svensson et al. 2009). Apart from dividing the sustainability concept into different criteria and indicators and to estimate their states and trends using verifier variables (step 5), it is necessary to compare the state and trends of indicators with norms (Lammerts van Bueren and Blom 1997) or performance targets (Villard and Jonsson 2009) (step 4). Defining the acceptable habitat loss for biodiversity maintenance is one example (Angelstam et al. 2004). Examples of appropriate tools for evaluation of biodiversity conservation are regional gap analysis and habitat suitability modeling (Angelstam et al. 2011a). Such assessments provide necessary input for policy decisions and landscape planning processes by different actors.

Assessment thus implies policy implementation research, which is about what develops between the establishment of an apparent intention to do something according to an agreed policy, or to stop doing something, and the ultimate impact of action on landscapes (O’Toole Jr. 2000; Sabatier 1986; Rauschmayer et al. 2009). Following Rauschmayer et al. (2009), it is necessary to understand the policy creation process, the outcomes of the implementation process of policy in terms of outputs such as rules, norms and planning tools, management, and finally the consequences on the ground in both ecological and social systems. Assessment also involves studies about stakeholders’ understanding, ability to act and willingness to act (Lundquist 1987) to make a diagnosis of policy implementation processes. The results of the assessments of processes in ecological (Angelstam et al. 2013e) and social systems (Axelsson et al. 2013b) should be communicated among stakeholders involved in decision-making processes at strategic, tactical, and operational levels.

#### Step 7. Comparisons and Syntheses

Once the six previous steps have been replicated in a suite of multiple case studies designed to sample a gradient in landscape history or governance (see step 1), comparative studies and meta-analyses can be used to generate and test hypotheses, and eventually draw conclusions. This is analogous to comparative politics at the level of countries and regions (Landman 2003). These individual case studies also provide depth (Merriam 1988). Finally, long-term studies can be made (Putnam et al. 1993). Such a triangulation approach employing several research approaches enhances production of knowledge about social–ecological systems in different contexts in terms of landscape history and governance arrangement. Similarly, comparison of multiple problem-solving learning processes contributes to the synthesis of tacit local knowledge to produce more generalized and explicit knowledge. Data on indicators for different criteria and knowledge of associated performance targets allow assessment and comparison of the level of different sustainability dimensions, from local to regional and transnational levels. Ultimately, an accounting system for landscape sustainability (Weaver and Rotmans 2006), which visualizes data for example by using maps (Axelsson et al. 2013a) can be used to improve the understanding about states and trends of different sustainability criteria by stakeholders at multiple levels. Such information forms the basis for transparent communication with and among decision-makers and stakeholders. Knowledge about the status and trends of sustainability is thus a necessary prerequisite needed for steering the development towards sustainability (Lee 1993). However, it is not sufficient. Additionally, a range of potential social actions (sensu Weber 1922; Parsons 1949) needs to be understood (see an example in Angelstam et al. 2013e).

### Collaboration Among Academic and Non-academic Actors

To realize the vision of SD towards sustainability in landscapes requires new knowledge and dissemination of existing experiences, representing both bridges and barriers to policy implementation. By contrast, research generally has a disciplinary character, including one individual, or a team of researchers, producing knowledge about increasingly specialized research topics. As a consequence, research does not always solve real world problems (European Commission 2005). Proposed approaches to resolve this issue include the following three levels of integrative research (Tress et al. 2006). Multidisciplinary research is when researchers from different disciplines work on a common theme but publish individually. Interdisciplinary research means that researchers from different disciplines try to build an interface towards a real world problem using their respective disciplines. Transdisciplinary research or knowledge production is where also real world stakeholders are included in the research process (Hirsch Hadorn et al. 2008). Transdisciplinary approaches thus require collaboration among academic and non-academic stakeholders. However, this brings new challenges to researchers, their networks, academia, and donors as well as to all other involved stakeholders (Gibbons 1999; Brulin and Svensson 2012; Angelstam et al. 2013a). Empirical studies about stakeholder collaboration, adaptive governance and management, as well as collaborative learning, indicates that these are crucial. Collaboration among researchers and practitioners is thus a critical component of the proposed seven-step framework to transdisciplinary research about SD and sustainability in addressing SD and sustainability (Daniels and Walker 2001); for a detailed example see Axelsson et al. (2013b).

### Communication with and Dissemination to Society

Dissemination of new knowledge at the research-policy-practice interface (Weaver and Rotmans 2006) is critical. As a consequence, frameworks for encouraging changed human behavior have been proposed regarding policy-makers, children and the general public (Defra 2005). Promoting environmental awareness through environmental education is one approach (Kopnina 2012). This requires the study of individuals’ environmentally significant behavior (Gardner and Stern 1996; Stern 2000). Different ways can be used with in the seven-step framework to attract attention and increase public awareness among societal actors interested in the development of sustainable landscapes. It is important to include sufficient resources to secure this expertise.

The first way is to share results from multiple-case studies using different media. As soon as the volume of relevant material grows from selected landscapes, journalists in different countries, who communicate the information to societal actors, are provided with press-releases, invited to press-conferences, seminars or workshops to share the knowledge (Frater 2011). The second way is to use the material gathered at the site in condensed form as educational or informational projects. The third way is to organize seminars and traveling workshops with diverse groups of societal actors to discuss specific topics within the wider context of landscape sustainability.

A logical follow-up would be to use knowledge gathered in multiple case studies to initiate long-term projects such as books or TV programs dealing with SD and sustainability of landscapes. Such projects can be seen either as historical records of a developing process or as ways to raise public awareness of a specific topic. Use of different communication strategies and platforms is crucial both for communication of existing knowledge among different societal sectors, and for bridging cultural barriers between countries, which is often a challenge for efficient communication and transparent information exchange. As the media landscape changes, so must these long-term projects be prepared to follow stakeholders and users to the platforms where they choose to seek new data and background information. Additionally, to assure involvement of non-academic actors in problem-solving, we argue in favor of selecting case study landscapes that ideally also contain units for research, education, and communication with society in general (Haberl et al. 2006; Elbakidze et al. 2012). Current research about the general awareness of the ecological and climatic challenges facing the world indicates that there is a better chance of a positive impact on the general public with best practice cases showing how individuals or groups can work for sustainability, rather than problem oriented publications.

## Discussion

### Linking Human Sciences, Natural Sciences, and Stakeholders

Applying the seven-step framework to knowledge production and learning for sustainable landscapes is an integrative (Tress et al. 2006) and transdisciplinary (Hirsch Hadorn et al. 2008) approach to research. This means researchers representing human sciences (i.e., humanities and social sciences) and natural sciences (see Snow 1993; Bloemers et al. 2010), as well as relevant non-academic actors, practice a collaborative learning process to solve complex natural resource issues (Daniels and Walker 2001; Van Paassen et al. 2011). Studying the implementation of ecological, economic, social, and cultural sustainability on the ground, and governance processes at multiple levels, means that landscapes are viewed as integrated social and ecological system that includes both place and space (Grodzynskyi 2005). The term landscape has several roots (Wiens et al. 2007), which encompass biophysical natural, anthropogenic, and perceived immaterial dimensions (Angelstam et al. 2013d). We view the different interpretations of the landscape concept as a suite of theoretical frameworks and practical tool to design and carry out multiple case studies for comparative transdisciplinary research of large spaces and places as social–ecological systems (see also Angelstam et al. 2013d).

Implementing the research program presented in Angelstam et al. (2007) we have applied the seven steps framework methodology in nine landscapes (Table 1). We view landscape case studies in the European continent’s steep gradients as a natural experiment (sensu Diamond 1986). While European ecoregions form broad longitudinal bands (Mayer 1984), there are distinct gradients between Europe’s East and West. This applies to ecological systems, which are more intact towards the north and the east (Lehtinen 2006; Edman et al. 2011). It also applies to social systems in terms of the gradient between western democratic market economies versus countries in transition from autarchic, centrally planned economies towards a market economy (Berend 1986; Chirot 1989; Janos 1989).

Keeping the zonal environmental conditions similar by focusing on Europe’s boreal and temperate forest biomes, the landscape case studies were made in gradients that represent variation in two main dimensions. The first was the history of land use ranging from areas with near-natural landscapes in the periphery of economic development to areas with a long history of landscape use and management. As proxy variables we used the gradient from occurrence of large intact landscapes to ecoregions with different levels of vulnerability, which is linked to gradients in landscape history (see Angelstam et al. 2013b). The second was the way governance is carried out using regional political divisions linked to the fault lines of political culture (Katchanovski 2006), or even termed civilizations (sensu Huntington 1997). These gradients can be simplified as a table with the two dimensions. Table 1 provides an overview of our studies carried out with this logic.

The results from studies applying the different steps in different case studies stress the need for bridging gaps between policy about SD and sustainability in both the European continent’s East and West (Borgström et al. 2006; Sandström et al. 2006; Lazdinis et al. 2007). With a pragmatic attitude to methods and disciplines the seven-step framework provides an approach to support the development of knowledge-based dialogue.

### Barriers and Bridges to Problem-Solving Research

The motivation behind our attempt to develop research that bridges gaps between disciplines on the one hand, and academia and practice on the other, was to enhance different stakeholders’ focus on all pillars of sustainability. Ultimately, the concerns behind policies on SD as a societal process and sustainability as outcomes on the ground have roots in the conservation of ecosystem composition, structure and function (Noss 1990) as natural capital and foundation for the human endeavor (Neumayer 2010). This applies to natural and semi-natural terrestrial and aquatic ecosystems in forest landscapes (Angelstam et al. 2004; Loucks and Gladwell 2009; Villard and Jonsson 2009), cultural landscapes (Birks et al. 1988; Angelstam 2006; Elbakidze and Angelstam 2007) and urban systems (Tzoulas et al. 2007). Using a holistic approach, ecosystem ecology has demonstrated how energy and nutrients depend on structure and composition of abiotic and biotic dimensions (Odum 1953). Already Odum (1959) was explicit about the role of functional ecosystems for social and cultural systems of humans. Methods to estimate the human footprint on ecosystems, and the usefulness of ecosystems to humans (e.g., MEA 2005; Kumar 2010), contributed to making ecosystem knowledge and understanding a part of policy developments in many sectors.

The lens of ecological sustainability involves the key challenges in support of policy development and application of measuring and communicating the state and trends of ecosystems as natural capital to societal stakeholders from different sectors at different levels of governance. In this process biocentric interfaces such as biodiversity (Wilson 1988) and anthropocentric interfaces such as the biophilia hypothesis (Kellert and Wilson 1993) and ecosystem services (Costanza et al. 1997; MEA 2005; Kumar 2010) are currently used. The term natural capital is the economic metaphor for the limited stocks of physical and biological resources in the Earth’s ecosystems (MEA 2005). Biodiversity in a particular area consists of: (1) the variety of life forms at various levels of organization (i.e., genetic, species, population, community); (2) the interactions between and within them; (3) the associated ecological processes needed to sustain them. Wilson (1984) used the term biophilia to describe human’s affinity to other forms of life linked to the structure of our brain’s basic mental facilities and tendency to focus on life and lifelike processes. The term ecosystem services (or goods and services) focuses on the direct and indirect provisioning, regulation, supporting and cultural benefits of ecosystems to human well-being (MEA 2005). Thus, ecological sustainability constitutes the core of both the biodiversity and ecosystem service concepts.

Economic sustainability is becoming increasingly complex, and involves controversies among different schools of valuation. Traditionally, neoclassical economists assume that a market-based economy will secure efficient allocation of natural resources among competing uses, and provide signals (prices, profits, rents) to different actors (firms, households, governments), which then respond in predictable ways. However, markets can also fail in this allocation of resources (e.g., Hanley 1998). For example, to use and degrade water, clean air or biodiversity as natural capital represents an external cost. This has provided legitimacy for governmental intervention. The total economic value concept (Merlo and Croitoru 2005) is another attempt towards remediation based on market valuations (market values, markets for substitute products and potential market values). Indeed businesses may consider the total economic value of intangible utilities. A good reason for this is profit-maximization due to expected good-will from environmental and social programs and thereby increased demand for the products (Djurberg et al. 2004). However, as argued by Gomez-Baggethun and Ruiz-Perez (2011) the commodification of ecosystem services may have counterproductive effects in the long term for natural capital and equity of access to ecosystem benefits. Additionally, social choice techniques can be used to elicit and identify owners’ values, preferences, and attitudes associated with their use of landscapes (Kearney and Kaplan 1997; Richnau et al. 2013). Intrinsic values, which may stem from traditions and cultures with rights-based belief systems (Spash and Simpson 1994) may also have importance. Such belief systems and organizations can be included into business ethics in terms of political economic organizations (sensu Söderbaum 2000).

Social sustainability is the third pillar of sustainable development processes. Social capital includes human relations and networks (Bourdieu 1986; Putnam 2000; Florida 2012) as a basis for social learning to allow or empower local people to steer their own development towards a desired state (Leeuwis and Pyburn 2002; Keen et al. 2005; Wals 2009). A policy area that aims at dealing with how to build social capital is rural development in landscapes with low human population (Lehtinen 2006; FORMAS 2007; Waldenström and Westholm 2009). This policy area aims to enhance coordinated and locally adapted ways to address pressing economic, social, and environmental problems in rural areas (Van der Ploeg et al. 2000; Moseley 2003). Additionally, cultural heritage can support rural development and includes both tangible parts, such as human built objects, environments and landscapes, and intangible parts, such as kinship relations, ethnic identity, practices, representations, expressions, knowledge and skills (e.g., Culture 21 2011).

## Conclusions

Applying the seven-step framework presented in this study in multiple social–ecological systems revealed different kinds of challenges. (1) There is insufficient knowledge about how the policy vision of sustainable development and sustainability in landscapes can satisfy societal needs in terms of goods, services and intangible values provided by landscapes, and how economic, ecological, social and cultural dimensions of sustainability can be realized. (2) Existing knowledge is often not communicated across different societal sectors due to sector-specific management and limited participation. (3) Cultural and language barriers among countries are a challenge for efficient communication and transparent information exchange. (4) The capacity of ecological systems is limited, i.e., we cannot satisfy all demands in all places. Physical planning is therefore needed, including zoning approaches and assessment of sustainability outcomes at multiple spatial scales. However, this is an unresolved governance challenge, especially in landscapes with many owners and user of lands and waters, and many stakeholders. (5) Another challenge is to bridge different actor’s understanding of their situations and needs in space and time (Soloviy and Keeton 2009; Sandström et al. 2011). (6) The challenge of going from experiences to learning and knowledge production locally, nationally and in international networks among different initiatives takes time and involves major transaction costs (Axelsson et al. 2013b). (7) Finally, evaluation is a key challenge (Leavy 2011). This applies in particular to the social learning process (Axelsson et al. 2013b).

To realize locally adapted visions for sustainable landscapes there is thus an urgent need to disseminate holistic, catchment-based or landscape level knowledge as a compass to support the gyroscope in terms of SD process (Lee 1993, Gibbons et al. 1994). Case studies are probably one of the best means to effectively achieve this (Gill 2011). Comprehensive and constructive evaluation of SD and sustainability of social–ecological systems in general, or existing landscape approaches and their on-the-ground application requires both a good dialogue with everyday practices in civil, private, and public sectors, and transdisciplinary research carried out by researchers who know human and natural science methods, and who can and are willing to collaborate. However, collaboration is a challenge if there are unequal conditions among stakeholders and actors. Trust and trustworthiness among collaborating actors takes time to build, and are often based on concrete results and shared benefits (see Axelsson et al. 2013b).

To conclude, we value highly the experiences gained from leaving the disciplinary academic researchers’ comfort zone (see Palmer 2012) and instead focusing on knowledge production and collaborative learning that includes both researchers and practitioners (e.g., Axelsson et al. 2013b). Our experience from developing and applying the seven-step framework is that in-depth exchange among researchers from different disciplines, and stakeholders at multiple levels, is a promising approach to bridge both cultures, and sectors using different landscape goods, services and intangible values, for the long-term success of SD towards sustainability.

## Electronic supplementary material

Below is the link to the electronic supplementary material.

Supplementary material 1 (PDF 276 kb)
