# Supplementary material for: Knowledge Production and Learning for Sustainable Landscapes: Seven Steps Using Social–Ecological Systems as Laboratories
Source: Ambio. 2013 Mar 10;42(2):116–28. doi: 10.1007/s13280-012-0367-1 (PMC3593032; doi:10.1007/s13280-012-0367-1)
Supplement: Supplementary file 1 — Supplementary material 1 (PDF 276 kb) [file 13280_2012_367_MOESM1_ESM.pdf]

Electronic Supplementary Material

Knowledge production and learning for sustainable landscapes: seven steps using social-ecological systems as laboratories

Per Angelstam, Marine Elbakidze, Robert Axelsson, Malcolm Dixelius, Johan Törnblom

References to studies made in the nine landscape case studies listed in Table 1

Ångermanälven catchment and Vilhelmina Model Forest (NW Sweden)

Angelstam, P., J. Törnblom, E. Degerman, L. Henrikson, L. Jougda, M. Lazdinis, J.C. Malmgren, and L. Myhrman. 2006. From forest patches to functional habitat networks – the need for holistic understanding of ecological systems at the landscape scale. In *Farming, forestry and the natural heritage: towards a more integrated future*, ed. R. Davison, and C.A. Galbraith, 193-209. The Stationery Office: The Natural Heritage of Scotland Series.

Angelstam, P., M. Elbakidze, and J. Törnblom. 2010. Mountain rivers in northern Sweden as a natural resource - the need for an integrated landscape approach. In: *Europe's ecological backbone: recognising the true value of our mountains*, 108-109. Copenhagen: European Environmental Agency.

Axelsson, R., and P. Angelstam. 2011. Uneven-aged forest management in boreal Sweden: local forestry stakeholders' perceptions of different sustainability dimensions. *Forestry* 84 (5): 567-579.

Axelsson, R., P. Angelstam, and J. Svensson. 2007. Natural forest and cultural woodland with continuous tree cover in Sweden: How much remains and how is it managed? *Scandinavian Journal of Forest Research* 22: 545-558.

Elbakidze, M., P. Angelstam, C. Sandström, and R. Axelsson. 2010. Multi-stakeholder collaboration in Russian and Swedish Model Forest initiatives: adaptive governance towards sustainable forest management? *Ecology and Society* 15(2): 14.

Bergslagen region (south-central Sweden)

Andersson, K., P. Angelstam, R. Axelsson, M. Elbakidze, and J. Törnblom. 2012. Connecting municipal and regional level planning: analysis and visualization of sustainability indicators in Bergslagen, Sweden. *European Planning Studies*. doi: 10.1080/09654313.2012.722943.

Andersson, K., P. Angelstam, M. Elbakidze, R. Axelsson, and E. Degerman. 2013. Green infrastructures and intensive forestry: need and opportunity for spatial planning in a Swedish rural-urban gradient. *Scandinavian Journal of Forest Research* 28(2): 143-165.

Angelstam, P., J. Törnblom, E. Degerman, L. Henrikson, L. Jougda, M. Lazdinis, J.C. Malmgren, and L. Myhrman. 2006. From forest patches to functional habitat networks – the need for holistic understanding of ecological systems at the landscape scale. In *Farming, forestry and the natural heritage: towards a more integrated future*, ed. R. Davison, and C.A. Galbraith, 193-209. The Stationery Office: The Natural Heritage of Scotland Series.

Angelstam, P., K. Andersson, R. Axelsson, M. Elbakidze, B.-G. Jonsson, and J.-M. Roberge. 2011. Protecting forest areas for biodiversity in Sweden 1991-2010: policy implementation process and outcomes on the ground. *Silva Fennica* 45(5): 1111–1133.

Angelstam, P., R. Axelsson, M. Elbakidze, L. Laestadius, M. Lazdinis, M. Nordberg, I. Pătru-Stupariu, and M. Smith. 2011. Knowledge production and learning for sustainable forest management: European regions as a time machine. *Forestry* 84 (5): 581-596.

Angelstam, P., K. Andersson, M. Isacson, D.V. Gavrilov, R. Axelsson, M. Bäckström, E. Degerman, M. Elbakidze et al. 2013. Learning about the history of landscape use for the future: consequences for ecological and social systems in Swedish Bergslagen. *AMBIO* 42(2): 146–159.

Axelsson, R., and P. Angelstam. 2011. Uneven-aged forest management in boreal Sweden: local forestry stakeholders' perceptions of different sustainability dimensions. *Forestry* 84 (5): 567-579.

Axelsson, R., P. Angelstam, and J. Svensson. 2007. Natural forest and cultural woodland with continuous tree cover in Sweden: How much remains and how is it managed? *Scandinavian Journal of Forest Research* 22: 545-558.

Axelsson, R., P. Angelstam, L. Myhrman, S. Sädbom, M. Ivarsson, M. Elbakidze, K. Andersson, P. Cupa et al. 2013. Evaluation of multi-level social learning for sustainable landscapes: perspective of a development initiative in Bergslagen, Sweden. AMBIO 42(2): 241–253.

Elbakidze, M., P. Angelstam, C. Sandström, and R. Axelsson. 2010. Multi-stakeholder collaboration in Russian and Swedish Model Forest initiatives: adaptive governance towards sustainable forest management? *Ecology and Society* 15(2): 14.

Elbakidze, M., P. Angelstam, K. Andersson, M. Nordberg, and Yu. Pautov. 2011. How does forest certification contribute to boreal biodiversity conservation? Standards and outcomes in Sweden and NW Russia. *Forest Ecology and Management* 262: 1983-1995.

Elbakidze, M., K. Andersson, P. Angelstam, G.W. Armstrong, R. Axelsson, F. Doyon, M. Hermansson, J. Jacobsson et al. 2013. Sustained yield forestry in Sweden and Russia: how does it correspond to sustainable forest management policy? *AMBIO* 42(2): 160–173.

Lazdinis, M., and P. Angelstam. 2005. Functionality of riparian forest ecotones in the context of former Soviet Union and Swedish forest management histories. *Forest Policy and Economics* 7(3): 321-332.

Helgeå catchment and Kristianstad Vattenrike (south Sweden)

Elbakidze, M., T. Hahn, V. Mauerhofer, P. Angelstam, and R. Axelsson. 2013. Legal framework for biosphere reserves as learning sites for sustainable development: a comparative analysis of Ukraine and Sweden. *AMBIO* 42(2): 174–187.

Richnau, G., P. Angelstam, S. Valasiuk, L. Zahvoyska, R. Axelsson, M. Elbakidze, J. Farley, I. Jönsson et al. 2013. Multi-faceted value profiles of forest owner categories in South Sweden: the River Helge å catchment as a case study. *AMBIO* 42(2): 188–200.

Bialowieza forest (NE Poland)

Angelstam, P., and M. Dönz-Breuss. 2004. Measuring forest biodiversity at the stand scale – an evaluation of indicators in European forest history gradients. *Ecological Bulletins* 51: 305-332.

Blicharska, M., and P. Angelstam. 2010. Conservation at risk: conflict analysis in the Bialowieza Forest, a European biodiversity hotspot. *International Journal of Biodiversity Science, Ecosystems Services & Management* 6(1): 68-74.

Blicharska, M., P. Angelstam, H. Antonson, M. Elbakidze, and R. Axelsson. 2011. Road, forestry and regional planners' work for biodiversity conservation and public participation: a case study in Poland's hotspots regions. *Journal of Environmental Planning and Management* 54(10): 1373-1395.

Edman, T., P. Angelstam, G. Mikusinski, J.-M. Roberge, and A. Sikora 2011. Spatial planning for biodiversity conservation: Assessment of forest landscapes' conservation value using umbrella species requirements in Poland. *Landscape and Urban Planning* 102: 16-23.

Roberge, J.-M., and P. Angelstam. 2006. Indicator species among resident forest birds – a cross-regional evaluation in northern Europe. *Biological Conservation* 130: 134-147.

Roberge, J.-M., P. Angelstam, and M.-A. Villard. 2008. Specialised woodpeckers and naturalness in hemiboreal forests - deriving quantitative targets for conservation planning. *Biological Conservation* 141: 997-1012.

The Carpathian Mountains in Lviv region (W Ukraine)

Angelstam, P., and M. Elbakidze. 2006. Sustainable forest management in Europe's East and West: trajectories of development and the role of traditional knowledge. In: Parrotta, J., Agnoletti, M., Johan, E. (Eds.), *Cultural heritage and sustainable forest management: the role of traditional knowledge.* MCPFE Proceedings, part 2, 353-361.

Angelstam, P., M. Elbakidze, R. Axelsson, P. Čupa, L. Halada, Z. Molnar, I. Patru-Stupariu, K. Perzanowski, et al. 2013. Maintaining cultural and natural biodiversity in the Carpathian Mountain ecoregion: need for an integrated landscape approach. In *Integrating nature and society towards sustainability*, ed. J. Kozak, K. Ostapowicz, A. Bytnerowicz, and B. Wyżga. Dordrecht: Springer. 10.1007/978-3-642-12725-0_28.

Elbakidze, M., and P. Angelstam. 2013. Sustainable forest management from policy to landscape, and back again: a case study in the Ukrainian Carpathian Mountains. In *Integrating nature and society towards sustainability,* ed. J. Kozak, K. Ostapowicz, A. Bytnerowicz, and B. Wyżga. Dordrecht: Springer. doi: 10.1007/978-3-642-12725-0_23.

Elbakidze, M., and P. Angelstam. 2007. Implementing sustainable forest management in Ukraine's Carpathian Mountains: the role of traditional village systems. *Forest Ecology and Management* 249: 28-38.

Keeton, W.S., P. Angelstam, M. Baumflek, Y. Bihun, M. Chernyavskyy, S. M. Crow, A. Deyneka, M. Elbakidze, et al. 2013. Sustainable forest management alternatives for the Carpathian Mountain region, with a focus on Ukraine. In *Integrating nature and society towards sustainability,* ed. J. Kozak, K. Ostapowicz, A. Bytnerowicz, and B. Wyżga. Dordrecht: Springer. DOI: 10.1007/978-3-642-12725-0_24.

Törnblom, J., P. Angelstam, E. Degerman, L. Henrikson, T. Edman, and J. Temnerud. 2011. Catchment land cover as a proxy for macroinvertebrate assemblage structure in Carpathian Mountain streams. *Hydrobiologica* 673: 153–168.

Roztochya Biosphere Reserve (W Ukraine)

Baumann, M., T. Kuemmerle, M. Elbakidze, M. Ozdogan, V.C. Radeloff, N. Keuler, A. Prishchepov, I. Kruhlov, et al. 2011. Patterns and drivers of post-socialist farmland abandonment in Western Ukraine. *Land Use Policy* 2: 552–562.

Elbakidze, M., P. Angelstam, C. Sandström, N. Stryamets, S. Crow, R. Axelsson, G. Stryamets, and T. Yamelynets. 2013. Biosphere Reserves for conservation and development in Ukraine? Legal recognition and establishment of the Roztochya initiative. *Environmental Conservation.* doi: 10.1017/S0376892912000434.

Elbakidze, M., T. Hahn, V. Mauerhofer, P. Angelstam, and R. Axelsson. 2013. Legal framework for biosphere reserves as learning sites for sustainable development: a comparative analysis of Ukraine and Sweden. *AMBIO* 42(2): 174–187.

Stryamets, N., M. Elbakidze, and P. Angelstam. 2011. Role of non-wood forest products for local livelihoods in countries with transition or market economy: case studies in Ukraine and Sweden. *Scandinavian Journal of Forestry* 27(1): 74-87.

Kovdozersky Model Forest (Murmansk oblast, NW Russia)

Elbakidze, M., P. Angelstam, and R. Axelsson. 2007. Developing sustainable forest management in North-West Russia: experiences from three implementation projects. - *Forest Facts* 3. Forest Research at the Swedish University of Agricultural Sciences. 4 pp.

Elbakidze, M., P. Angelstam, and R. Axelsson. 2007. Sustainable forest management as an approach to regional development in the Russian Federation: state and trends in Kovdozersky Model Forest in the Barents region. *Scandinavian Journal of Forest Research* 22: 568-581.

Elbakidze, M., P. Angelstam, P., and R. Axelsson. 2012. Stakeholder identification and analysis for adaptive governance in the Kovdozersky Model Forest, Russian Federation. *The Forestry Chronicle* 88 (3): 298-305.

Priluzie Model Forest (Komi Republic, NW Russia)

Elbakidze, M., P. Angelstam, C. Sandström, and R. Axelsson. 2010. Multi-stakeholder collaboration in Russian and Swedish Model Forest initiatives: adaptive governance towards sustainable forest management? *Ecology and Society* 15(2): 14.

Elbakidze, M., P. Angelstam, K. Andersson, M. Nordberg, and Yu. Pautov. 2011. How does forest certification contribute to boreal biodiversity conservation? Standards and outcomes in Sweden and NW Russia. *Forest Ecology and Management* 262: 1983-1995.

Elbakidze, M., K. Andersson, P. Angelstam, G.W. Armstrong, R. Axelsson, F. Doyon, M. Hermansson, J. Jacobsson, et al. 2013. Sustained yield forestry in Sweden and Russia: how does it correspond to sustainable forest management policy? *AMBIO* 42(2): 160–173.

Pskov Model Forest (Pskov oblasts, W Russia)

Angelstam, P., and M. Dönz-Breuss. 2004. Measuring forest biodiversity at the stand scale – an evaluation of indicators in European forest history gradients. *Ecological Bulletins* 51: 305-332.

Elbakidze, M., P. Angelstam, C. Sandström, and R. Axelsson. 2010. Multi-stakeholder collaboration in Russian and Swedish Model Forest initiatives: adaptive governance towards sustainable forest management? *Ecology and Society* 15(2): 14.
